# Impact of Graphene Layers on Genetic Expression and Regulation within Sulfate-Reducing Biofilms

**DOI:** 10.3390/microorganisms12091759

**Published:** 2024-08-24

**Authors:** Vinoj Gopalakrishnan, Priya Saxena, Payal Thakur, Alexey Lipatov, Rajesh K. Sani

**Affiliations:** 1 Department of Chemical and Biological Engineering, South Dakota School of Mines and Technology, Rapid City, SD 57701, USA; vinojvino@gmail.com (V.G.); priya.saxena@mines.sdsmt.edu (P.S.); payal.thakur@mines.sdsmt.edu (P.T.); alexey.lipatov@sdsmt.edu (A.L.); 2 Data Driven Material Discovery Center for Bioengineering Innovation, South Dakota School of Mines and Technology, Rapid City, SD 57701, USA; 3 2-Dimensional Materials for Biofilm Engineering, Science and Technology, South Dakota School of Mines and Technology, Rapid City, SD 57701, USA

**Keywords:** corrosion, green fluorescent protein, *Oleidesulfovibrio alaskensis*, regulatory proteins, quorum sensing

## Abstract

Bacterial adhesion and biofilm maturation is significantly influenced by surface properties, encompassing both bare surfaces and single or multi-layered coatings. Hence, there is an utmost interest in exploring the intricacies of gene regulation in sulfate-reducing bacteria (SRB) on copper and graphene-coated copper surfaces. In this study, *Oleidesulfovibrio alaskensis* G20 was used as the model SRB to elucidate the pathways that govern pivotal roles during biofilm formation on the graphene layers. Employing a potent reporter green fluorescent protein (GFP) tagged to *O. alaskensis* G20, the spatial structure of *O. alaskensis* G20 biofilm on copper foil (CuF), single-layer graphene-coated copper (Cu-GrI), and double-layer graphene-coated copper (Cu-GrII) surfaces was investigated. Biofilm formation on CuF, Cu-GrI, and Cu-GrII surfaces was quantified using CLSM z-stack images within COMSTAT v2 software. The results revealed that CuF, Cu-GrI, and Cu-GrII did not affect the formation of the GFP-tagged O. *alaskensis* G20 biofilm architecture. qPCR expression showed insignificant fold changes for outer membrane components regulating the quorum-sensing system, and global regulatory proteins between the uncoated and coated surfaces. Notably, a significant expression was observed within the sulfate reduction pathway confined to dissimilatory sulfite reductases on the Cu-GrII surface compared to the CuF and Cu-GrI surfaces.

## 1. Introduction

Sulfur is considered to be the 10th most abundant element present in the natural habitat and can exist in various sulfide and sulfate minerals, including pyrite (FeS_2_) and gypsum (CaSO_4_) [1]. Sulfate-reducing bacteria (SRB) are strict anaerobes that thrive on sulfate and utilize it as a terminal electron acceptor by reducing it to hydrogen sulfide [2]. Microbes can easily transition from free-swimming states to surface-attached biofilms in response to diverse stimuli [3]. As these bacterial communities evolve, a matrix of extracellular polymeric substances (EPS) plays a crucial role in providing structural integrity and protection to the bacteria within the biofilm [4]. This matrix, composed of polysaccharides, proteins, and extracellular DNA, collectively forms a shield against environmental stress [4].

SRB can form biofilm when they encounter any metal surfaces, which leads to and promotes redox processes [5]. SRB biofilms pose significant disadvantages, particularly causing metal corrosion across industries, leading to acid mine drainage and oil spills from deteriorating pipelines and storage tanks [6]. This process, known as microbial induced corrosion (MIC), results in substantial economic losses and safety hazards [5]. Moreover, in biomedical settings, these biofilms pose severe limitations that include reduced heat transfer efficiency and medical device contamination. Low-cost anti-MIC approaches are frequently based on biocides, which have an adverse effect on surrounding organisms as well as the environment [7]. Therefore, an urgent need for protective coatings leads to a constant search for 2D and polymeric materials. SRB have developed several pathways to overcome copper toxicity, allowing them to survive and thrive in environments containing elevated copper concentrations. For instance, *Oleidesulfovibrio alaskensis* G20 exhibits copper resistance, with its corrosion behavior and mechanism well reported by many research groups [2,3]. *O. alaskensis* biofilm formation can act as a protective barrier against copper toxicity by physically limiting the penetration of copper ions into bacterial cells [4,5]. Further, the growth of bacteria is neither stimulated nor inhibited by graphene materials. The perceived bactericidal action of graphene is due to the presence of impurities in graphene [6,7].

Over the years, many commercial coatings such as polyaniline have been employed to mitigate MIC [8]. However, graphene demonstrates significant toxic effects in biofilms formation and acts as a wide antibacterial agent against the majority of bacteria [9,10,11,12]. Studies on graphene interactions with living cells, specifically bacteria, have produced findings that seem to conflict. According to recent studies, graphene promotes the formation of biofilms and bacterial growth [13,14,15]. Graphene is a promising candidate for biological, antifouling, and anticorrosion applications despite the lack of substantial research in these areas. A small number of papers have reported the implementation of two-dimensional (2D) coatings with anti-biofilm activity to evaluate the effects of MIC on 2D coated metal surfaces in SRB [16,17,18,19]. However, the results showed significant reduction in biofilm formation and MIC rates when 2D coatings were employed on metal surfaces. Conversely, the effect of graphene atomic layers on metal surfaces is still unclear in the aspects of microbial attachment and MIC [16,17]. Therefore, the effect of single and double layers of 2D material are an interesting area of study to unveil the microbial attachment and biofilm formation rates. A study by Chilkoor et al. showed that a single-layer hexagonal boron nitride coating on a copper surface serves as an effective barrier to MIC and reported approximately 91% corrosion inhibition in *Oleidesulfovibrio alaskensis* G20 with respect to other commercial coatings, whereas another study by Chilkoor et al. revealed that the single graphene layer enhanced the biogenic sulfide attack resulting in increased MIC [16,20]. However, the literature lacks the genotypic and phenotypic behavior of SRB, and the key responsible genes regulating biofilm formation/inhibition on 2D materials.

In this study, we used *O. alaskensis* G20 as a model Gram negative SRB due to its biofilm-forming capabilities and environmental relevance. This bacterium thrives in diverse environments, from marine sediments to hydrothermal vents, playing advantageous and disadvantageous roles in sulfur cycle and MIC, respectively. Despite its multifaceted ecological function, the genetic regulation of this organism on 2D materials remains unexplored. This study will explore the regulatory genes associated with crucial metabolic pathways which include sulfate metabolism, carbon metabolism, signaling pathways and biofilm-forming genes to deduce their involvement in biofilm formation on a 2D graphene-coated copper surface. Furthermore, the study aims to demonstrate a better and comparable understanding of biofilm formation and the respective expression of genes involved in biofilm formation when cultivated on both coated and uncoated graphene surfaces.

## 2. Materials and Methods

### 2.1. Bacterial Inoculation and Cultivation

The *O. alaskensis* G20 cells for the experiment were anaerobically batch-cultured in 150 mL serum bottles in LS4D media of pH 7.2, with a working volume of 100 mL at 30 °C and 150 rpm [21]. The composition of LS4D media is shown in Table S1. The serum bottles for preparing the seed culture with 100 mL LS4D media were capped with rubber septa and sealed with aluminum caps. The prepared media was autoclaved at 121 °C for 15 min. After cooling to room temperature, nitrogen gas was purged at 10 psi for 12 min to provide an anaerobic environment within the serum bottles. The seed culture of *O. alaskensis* G20 cells was prepared by inoculating the cells from the glycerol stock (maintained at −80 °C) and was grown at 30 °C and 150 rpm for 4 days. Further experiments were performed in the batch reactor (CDC biofilm reactor, Biosurface technologies corporation, Bozeman, MT, USA) in an anaerobic chamber (COY lab products). Two reactors with 250 mL of media were autoclaved and purged with ultrapure nitrogen gas (to make the reactor anaerobic) for 30 min each. These reactors were transferred to the anaerobic chamber and were fixed with Cu, GrI, and GrII foil in the rods present in the reactor itself. One reactor consists solely of bare Cu foil; the second reactor contains graphene-coated foils. The reactors containing the autoclaved media with desired Cu GrI and GrII foils were inoculated with 10% (*v*/*v*) *O. alaskensis* G20 seed culture at the late-exponential phase (Day 3). The experiment was performed over a period of 12 days and statistical triplicates were used.

### 2.2. Graphene Growth

Graphene was synthesized via copper-catalyzed decomposition of methane, CH_4_, in a homebuilt CVD system. Copper substrates of 10 mm × 25 mm were prepared from a roll of 25 µm thick polycrystalline copper foil (99.8%+, Thermo Scientific, Waltham, MA, USA) and cleaned by soaking in glacial acetic acid (Certified ACS, Fisher Scientific, Waltham, MA, USA) for 5 min, followed by a rinse with deionized water, then isopropyl alcohol (ACS Grade, ≥99.5%, Fisher Scientific), and blown dry using N_2_ gas. The prepared pieces of foil were put into the 1 inch inner-diameter quartz tube of the horizontal tube furnace (Lindberg/Blue M). The CVD chamber was pumped down to a system vacuum of ~7 mTorr, then flushed with 300 sccm of Ar gas (ultra-high purity, 99.999%) for 3 min and vacuumed again. The cycle was repeated for a total of three flushes to remove oxygen from the chamber. Then, the gas flow rate was adjusted to 50 sccm of H_2_ (ultra-high purity, 99.999%) and 200 sccm of Ar for the thermal annealing of copper. The furnace was heated to 1000 °C over 40 min and held at 1000 °C for 20 min to anneal the Cu substrate and remove residual oxides. After that, the Ar gas was turned off, and CH_4_ gas (ultra-high purity, 99.999%) was introduced to the system at a rate of 270 sccm, resulting in the total pressure of the system being ~170 mTorr. The deposition at 1000 °C lasted for 15 min, after which the furnace was allowed to cool down to room temperature (~120 min) while methane and hydrogen gases were kept flowing to prevent oxidation. The copper foils with deposited graphene were removed from the system and subjected to visual examination and Raman spectroscopy. Figure S1a shows a photograph of several copper substrates coated with monolayer graphene prepared for this study. Figure S1b shows an exemplar of Raman spectra for one of the graphene samples, where the intensity of the 2D-band peak (2681 cm^−1^) is ~2 times higher than the intensity of the G-band peak (1590 cm^−1^), confirming that the graphene is a double layer.

### 2.3. Construction of the O. alaskensis G20 (pBMC6 pC3::gfp) Strain

The *gfp* gene was fused with the constitutive promoter pC3 of the c3 cytochrome, to establish the strain *O. alaskensis* G20 (pBMC6pC3::*gfp*) followed with the same procedure as previously reported [22]. To obtain *gfp*-*O. alaskensis* G20, pBMC6pC3::*gfp* plasmid was transformed into *O. alaskensis* G20 by electroporation as described in [23]. Briefly, approximately 1000 ng of plasmid DNA was added to the cells and mixed thoroughly. The resultant mixture was then transferred to an electroporation cuvette with a 1-mm gap (Molecular BioProducts, San Diego, CA, USA). Electroporation was carried out using the following parameters on an ECM 630 electroporator by BTX, Genetronix, San Jose, CA, USA: 1500 volts, 250 ohms, and 25 microfarads. The electroporated cells were allowed to recover in 5 mL of LS4D medium overnight at 32 °C. Aliquots of electroporated cell colonies were mixed with LS4D agar media and then poured into petri dishes to solidify. Transformation efficiency was expressed as the number of kanamycin- and thiamphenicol-resistant colonies seen after 12 days of incubation at 30 °C in the anaerobic chamber. *O. alaskensis* G20 expressing GFP cells were directly spotted onto microscope glass slides and mounted with a coverslip. Images and GFP fluorescence were acquired using a confocal laser microscope under Argon. GFP was excited at 488 nm, and emission was recorded at 550 nm.

### 2.4. Sample Preparation for Confocal Microscopy

Biofilms formed on Cu, GrI and GrII foils were taken aseptically from the CDC reactor on the 12th day and washed carefully with anaerobic 30 mM PBS (pH 7.2) 3 times to remove the planktonic cells and media components. 1 µL Concanavalin A (ConA) (Alexa Fluor^®^ 647, Life Technologies, ThermoFisher Scientific, Waltham, MA, USA) conjugates dissolved in 1 mL of 2X saline sodium citrate buffer was used to mark glycosidic residues in the *gfp-O. alaskensis* G20 biofilm exopolysaccharide layer. The biofilm-covered surfaces were completely covered with the resulting stain mixture and were kept for incubation in the dark at 30 °C for 30 min. Following incubation, the excess stain was removed with 2X Saline Sodium Citrate buffer. After staining, the respective foils were visualized under a confocal microscope (Nikon, Nishio, Japan) using an x60 water immersion objective. The GFP-tagged cells and ConA-stained regions were first imaged separately to control for any channel-to-channel dispersion. The excitation/emission wavelengths for GFP and ConA visualization were 488 nm/550 nm and 561nm/650 nm, respectively. Six random locations were scanned on each Cu, GrI and GrII foil sample. In order to determine the biofilm thickness, and for 3D visualization and analysis, five z-stacks were obtained for each condition.

### 2.5. Crystal Violet Staining for Bacterial Biofilm Grown on Metal Surfaces

The CuF, GrI and GrII foils were removed from the bioreactor on the 4th, 8th and 12th days and gently rinsed with anaerobic PBS (pH 7.2) to remove non-adherent cells. The total number of adhered cells within the biofilm matrix was quantified using the crystal violet assay, as previously described [24]. Briefly, the foils were placed in a 24-well microtiter plate and were gently rinsed with sterile distilled water to remove any planktonic cultures. A quantity of 1 mL of 0.1% (*w*/*v*) crystal violet solution (dissolved in sterile distilled water) was added to each well, ensuring foils were dipped completely and were incubated at room temperature (in the dark) for 15 min. The foils were then gently rinsed three times with distilled water to eliminate any unbound crystal violet and allowed to air dry completely. To elute the bound crystal violet, the foils were washed with 1 mL of 95% ethanol, and the optical density (OD) was measured at 570 nm to quantify the stained biofilm matrix.

### 2.6. Colony Counting Method

A suspension of OAG20 cells in 1 mL of PBS buffer (pH 7.2) with an OD_600_ of 0.5, corresponding to 10^8^ CFU/mL, was used as a standard to evaluate cell counts on surfaces. Biofilm cells from the sample surfaces were gently scraped and suspended in 1 mL of PBS. The turbidity of the suspended cells was measured at OD_600_. To eliminate interference from non-cell particles, such as copper sulfide, the suspension was serially diluted five-fold. A 100 µL aliquot of the diluted suspension was then spread on culture plates containing LS4D media with 2.5% *w*/*v* agar and maintained anaerobically at 30 °C. After 72 h in an anaerobic chamber, the colonies formed were quantified using the colony counting method. The mean values were normalized with the serial dilutions, and statistical standard deviations were included.

### 2.7. RNA Extraction

The RNA extraction was carried out by harvesting the *O. alaskensis* G20 biofilm at the late exponential phase (Day 12). The biofilm pellet was collected and washed down three times with anaerobic 30 mM phosphate buffer saline (pH 7.2), to remove any unwanted media components that may hamper the further procedure of extraction. After washing, the pellets were transferred to sterile 2 mL microcentrifuge tubes for further extraction steps. Microbial RNA was isolated using the TRIzol reagent kit (Invitrogen, Carlsbad, CA, USA) according to the manufacturer’s protocol [25,26]. The extracted RNA was eluted in 20 µL nuclease-free water. Later, the RNA was purified using the RNA clean and concentrator Kit (ZymoResearch, Irvine, CA, USA, Catalog #R1019). The purified RNA was eluted in 10 µL nuclease-free water. The concentration and RNA integrity number (RIN) of the purified RNA was determined using NanoDrop 2000 (Thermofisher, Waltham, MA, USA), and Bioanalyzer 2100 (Agilent, Santa Clara, CA, USA).

### 2.8. cDNA Synthesis and qPCR

The RNA was extracted and purified as described in the previous section on RNA isolation. cDNA synthesis was performed using the QuantiTect Reverse Transcription Kit (Qiagen, Germantown, MD, USA). Subsequently, RT-qPCR was performed in a QuantStudio 3 real-time PCR system (Thermo Fisher Scientific, Waltham, MA, USA) using the Maxima SYBR Green/ROX qPCR Master Mix (Thermo Fisher Scientific, Waltham, MA, USA) in a 96-well plate. Gene-specific primers used for RT-qPCR are shown in Table S2. Recombinase A (*recA*) was used as an internal gene standard for PCR amplification and data normalization. Normalized fold changes of the relative expression ratio between the biofilm control (Cu) and GrI and GrII samples were quantified by the 2^−ΔΔCT^ method [27]. All experiments were performed in triplicate using independent samples, and their mean value and standard error of the mean were calculated. The genes selected are involved in key processes such as carbon metabolism, dissimilatory sulfate reduction, biofilm formation, and signaling pathways. These genes were chosen based on their critical roles in metabolism and their relevance to biofilm-related studies documented in the literature. The selected genes include *LuxP* (Dde3311, binding periplasmic protein), HK (Dde3717, sensor histidine kinase), Ps (Dde3253, polysaccharide biosynthesis protein), *RpoN* (Dde2052, sigma 54 transcription factor), *sat* (sulfate adenyl transferase, Dde2265), and *dsrA* (dissimilatory sulfite reductase, Dde0526).

### 2.9. Statistical Analysis

The data were analyzed using Graph Pad Prism (version 9) computer software. Tukey’s post hoc test was used to determine the differences between the coated and uncoated samples.

## 3. Results and Discussion

### 3.1. Effects of Graphene Layer on gfp-O. alaskensis G20 Adhesion and Biofilm Formation

**Figure 1:**
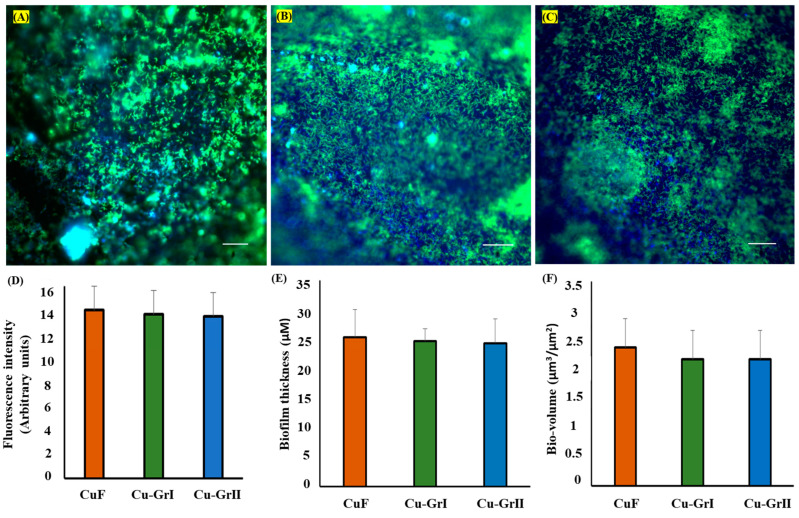
Confocal laser scanning microscopy (CLSM) images of 12th day O. alaskensis G20 biofilms grown on (**A**) CuF, (**B**) Cu-GrI, and (**C**) Cu-GrII surfaces. (**D**) Comparison of fluorescence intensity of ConA-stained EPS matrix secreted within the biofilm on CuF, Cu-GrI, and Cu-GrII. (**E**) Biofilm thickness, and (**F**) bio-volume, measured using CLSM z-stack images. *p* < 0.05 significance was determined by comparing the uncoated with coated surface. Error bars represent the standard deviation of the data set (*n* = 5).

We studied the adherence of the *gfp*-*O. alaskensis* G20 to copper surface (CuF), single layer graphene-coated surface (Cu-GrI), and double layer graphene-coated surface (Cu-GrII), in a CDC biofilm reactor for 12 days. The biofilm formation of *gfp*-*O. alaskensis* G20 on the CuF, Cu-GrI and Cu-GrII surfaces is shown in (Figure 1A–C). The adhesion competence of *gfp*-*O. alaskensis* G20 is similar to that seen with biofilms developed on CuF surfaces, demonstrating that the presence of graphene layers in Cu-GrI and Cu-GrII samples had no impact on the early attachment features of *gfp*-*O. alaskensis* G20. The detection of colonies expressing pBMC6pC3-encoded GFP with sufficient intensity by confocal laser scanning microscopy (CSLM) indicates that GFP is stably maintained in significant populations of *O. alaskensis* G20 on both coated and uncoated surfaces. However, the fluorescence intensities were observed to be similar on both the coated and uncoated surfaces. After 12 days of incubation, the formation of the EPS matrix in GFP-*O. alaskensis* G20 biofilms was observed. ConA labeling, combined with COMSTAT analysis and CLSM imaging, enabled quantification of the EPS within the biofilm matrix and provided a visual representation of the EPS matrix’s impact on CuF, Cu-GrI, and Cu-GrII surfaces. It has been previously reported that bacterial EPS matrix composition varies significantly depending on the environment and the surface [28]. Fluorescence intensity studies demonstrated that EPS architectures were not impacted by the graphene surfaces throughout the biofilm development process on CuF, Cu-GrI, and Cu-GrII surfaces (Figure 1D). The biovolume and thickness of the Cu-GrI, Cu-GrII, and CuF biofilm surfaces showed no significant difference over the experimental period (Figure 1E,F).

### 3.2. O. alaskensis G20 Culturability and Biofilm Quantification on Surfaces

**Figure 2:**
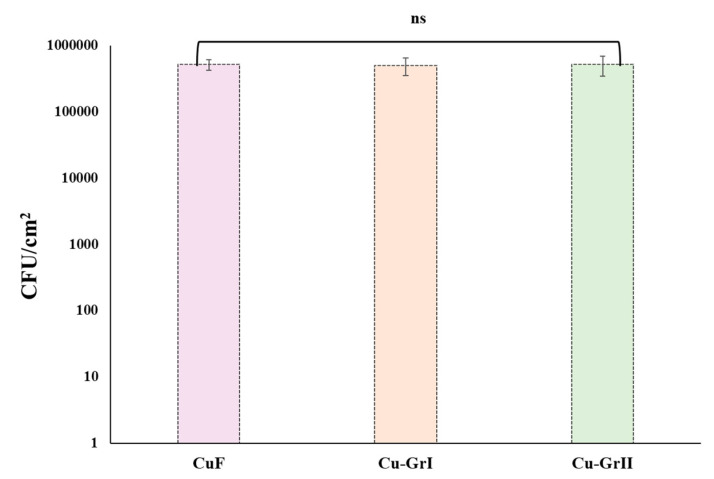
Bacteria cell counts within the biofilm matrix (logCFU/cm^2^) after 12 days of biofilm formation on CuF, Cu-GrI, and Cu-GrII surfaces. No statistically significant difference was observed on biofilm formation on the surfaces. The non-significant difference among the samples represented by “ns”.

The culturability of the *O. alaskensis* G20 biofilms formed on CuF, Cu-GrI and Cu-GrII surfaces was evaluated. Antibacterial activities of Cu-GrI and Cu-GrII materials did not show any significant difference when compared with the CuF surface. Our investigation indicates that *O. alaskensis* G20 viability showed no difference between coated and uncoated surfaces; *O. alaskensis* G20 concentration after 12 days incubation was 10^5^–10^6^ CFU mL^−1^ (Figure 2). Previous studies have reported potent antimicrobial properties of graphene-based materials, attributed to their ability to induce oxidative stress on a range of bacteria, including *E. coli*, *S. typhimurium*, *E. faecalis*, and *B. subtilis* [29,30,31].

**Figure 3:**
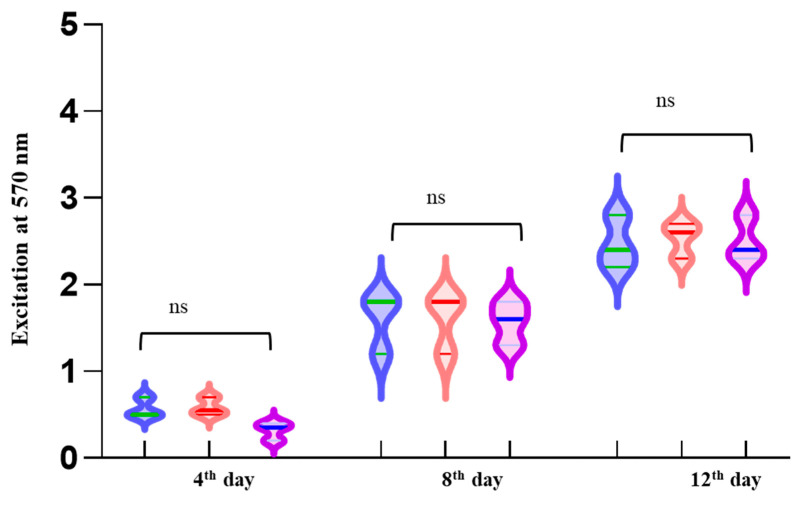
Crystal violet quantification of *O. alaskensis* G20 biofilms on 4^th^, 8^th^, and 12^th^ days grown on CuF (blue), Cu-GrI (orange), and Cu-GrII (pink) surfaces. Statistically, no significant difference was observed on biofilm formation on the surfaces (represented by “ns”).

The biofilms that developed on the surfaces of CuF, Cu-GrI, and Cu-GrII were substantially denser. Despite the consistently dense structure of the biofilms on Cu-GrI and Cu-GrII surfaces, no discernible differences were found between them (Figure 2). Furthermore, thick biofilm architecture on both coated and uncoated surfaces was consistent with the findings from previous studies. This indicates that graphene coatings have no bactericidal properties [32]. Similar results were obtained when the biofilm grown on the foils was quantified with crystal violet staining (Figure 3).

### 3.3. Expression Levels of Biofilm Genes in CuF, Cu-GrI, and Cu-GrII Surfaces

The formation of biofilms by *O. alaskensis G20* on CuF, Cu-GrI, and Cu-GrII surfaces was compared by analyzing the expression of six genes associated with carbon metabolism, sulfate reduction, biofilm formation, and signaling pathways. The selection of these genes is based on their involvement in fundamental survival metabolism and they are also crucial for biofilm formation as evidenced by reports in the literature where they showed significant expression levels [33,34,35].

**Figure 4:**
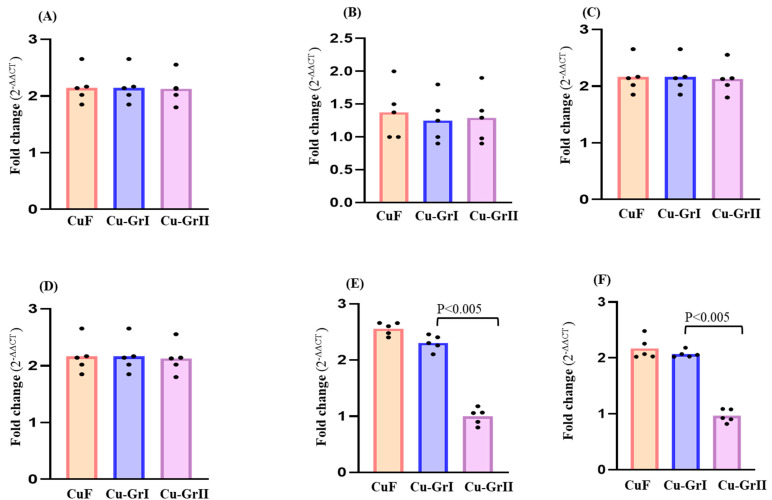
Biofilm gene expression levels (Cq values determined by qPCR) on various CuF, Cu-GrI, and Cu-GrII surfaces. (**A**) Polysaccharide (Ps), (**B**) sensor histidine kinase (HK), (**C**) binding periplasmic protein (LuxP), (**D**)sigma 54-dependent regulator (rPON), (**E**) dissimilatory sulfite reductase (dsrB), (**F**) sulfate adenylyl transferase (*sat*). The outliers are shown by dots (replicated samples with Cq values).

Sigma 54 plays a crucial role in activating genes that are responsible for cell survival under unfavorable conditions. Moreover, inactivation of these genes may affect cell-to-cell communication and reduce virulence, thus leading to aberrant flagellum development and motility [36]. The sigma 54-dependent two-component system is the primary mechanism by which *O. alaskensis* G20 regulates internal signals and adapts these signals into suitable cellular responses, which are involved in biofilm development. The expression of genes that maintain the integrity of periplasmic and outer membrane components is regulated by the sigma factors of the extracytoplasmic function protein family [37]. The fundamental regulatory network and stress response pathways in *O. alaskensis* G20 can quickly mobilize several gene expressions to adapt to different surfaces. In the current study, no visible variation in expression was observed for the genes quorum sensing *LuxP* (Dde3311, binding periplasmic protein), HK (Dde3717, sensor histidine kinase), Ps (Dde3253, polysaccharide biosynthesis protein), and *RpoN* (Dde2052, sigma 54 transcription factor) which contributes to the formation of the *O. alaskensis* G20 biofilm (shown in Figure 4). The metabolic pathways and interactions within biofilm architecture are activated by signaling molecules in sulfate-reducing bacteria [38,39].

The quorum sensing (QS) mechanism is used by SRB to control their biofilm forming behavior, while regulating metabolic activities. The QS system also contributes to sulfate reduction in SRB species, linking it to biofilm development and sulfate depletion [38,40]. The expression of the Ps, HK, LuxP, and RpoN genes, which are associated with quorum sensing and biofilm formation, showed that O. alaskensis G20 maintained its integrity and was not affected during biofilm development on the graphene layer, preserving the periplasmic and cytoplasmic components (Figure 4A–D). HK can modulate biofilm formation by regulating the expression of genes involved in EPS synthesis, flagellar motility, quorum sensing, and stress responses [41]. Additionally, HK has been shown to play a role in regulating the response of SRB to oxidative stress, which can occur during biofilm formation on metal surfaces [42,43]. However, our results indicate that the internal activities of the *O. alaskensis* G20 membrane were not affected by the graphene layer.

Among the studied genes, sulfate adenyl transferase Dde2265 (*sat*) and dissimilatory sulfite reductase Dde0526 (*dsrA*) showed variable expression. Both *sat* and *dsrA* on the Cu-GrII biofilm surface were upregulated by 3.0 times (*p*-value = 0.05) when compared to the CuF and Cu-GrI biofilm surfaces. In SRB, *sat* is a crucial enzyme, which is responsible for sulfate activation at the initial stage of sulfate reduction [44,45]. Previous studies showed that disruptions in the *sat* gene significantly reduced biofilm formation compared to the wild-type strain [44]. Also, the *sat* gene exhibited an important role in the expression of genes related to flagellar movement and exopolysaccharide production, both of which are crucial for the development of biofilms [45]. The expression of *dsrA* and *sat* transcripts may possibly reveal crucial roles in the metabolic state of *O. alaskensis* G20 (Figure 4E,F). Notably, *dsrA* transcript levels and sulfate reduction rates inside each individual cell could be intrinsically associated.

*O. alaskensis* has been studied for its ability to reduce sulfate (a common sulfur source) and oxidize organic substrates under anaerobic conditions. In the presence of copper, it can mediate the reduction of copper ions to metallic copper and/or the formation of copper sulfide minerals as part of its metabolic processes. However, the diffusion of copper ions through the single-layered graphene (SLG) and the formation of copper sulfide during biofilm growth were already well reported [16]. The study showed that the biogenic sulfide attack was five times higher with SLG than it was with bare Cu. The growth of bacteria is neither stimulated nor inhibited by graphene materials. The artifact on the bactericidal action of graphene may be due to the presence of impurities in graphene [16,46]. The proliferation of bacteria cannot be significantly restricted by the graphene film [47]. Similarly, although the graphene layers on copper (Cu) surfaces did not prevent *O. alaskensis* attachment and biofilm growth, they effectively restricted the biogenic sulfide attack. In our study, *O. alaskensis* biofilm formation on CuF and Cu-GrI surfaces after 12 days caused corrosion and leaching of metalloid ions, whereas Cu-GrII delayed the corrosion and contact of biofilm with the Cu surface. Previous studies reported that multilayer graphene serves as an impermeable barrier to protect the Cu surface from aggressive metabolites released by the biofilm [16].

## 4. Conclusions

A well-known mechanism of graphene coatings is their ability to block direct contact between sulfate-reducing bacteria and copper surfaces. Additionally, graphene coatings may prevent the leaching of metalloid ions that corrosive bacteria might otherwise utilize for their metabolism. The current work appears to be the first to show visualization, quantitative characterization, and real-time expression of the biofilms formed by an *O. alaskensis* G20 strain during the course of 12 days of incubation on a graphene-coated copper surface. This study highlights main observations regarding bacterial gene expression response to interactions in CuF, Cu-GrI, and Cu-GrII surfaces. It was revealed that the Cu-GrII surface differed in the expression of *O. alaskensis* G20 biofilm-forming genes during sulfate metabolism. The difference in the expression of the genes *sat* and *dsrA* is associated with the formation of biofilm on the Cu-GrII surface, suggesting the contribution of the sulfate reduction pathway to the corrosion characteristics of *O. alaskensis* G20. In our subsequent studies, understanding the molecular mechanisms underlying biofilm development and corrosion mechanisms will require additional investigation of the metabolome and gene silencing through CRISPR interference, particularly focusing on the carbon and sulfate metabolic pathways.

## Data Availability

Data are contained within the article and Supplementary Materials.

## Supplementary Materials

The following supporting information can be downloaded at: https://www.mdpi.com/article/10.3390/microorganisms12091759/s1, Figure S1: Raman spectra of monolayer graphene on a copper substrate; Table S1: LS4D media components; Table S2: Sequences of oligonucleotide primers used for qPCR.
