# Supplementary material for: Impact of Graphene Layers on Genetic Expression and Regulation within Sulfate-Reducing Biofilms
Source: Microorganisms. 2024 Aug 24;12(9):1759. doi: 10.3390/microorganisms12091759 (PMC11433944; doi:10.3390/microorganisms12091759)
Supplement: Supplementary file 1 [file microorganisms-12-01759-s001.zip › microorganisms-3131378-supplementary material.pdf]

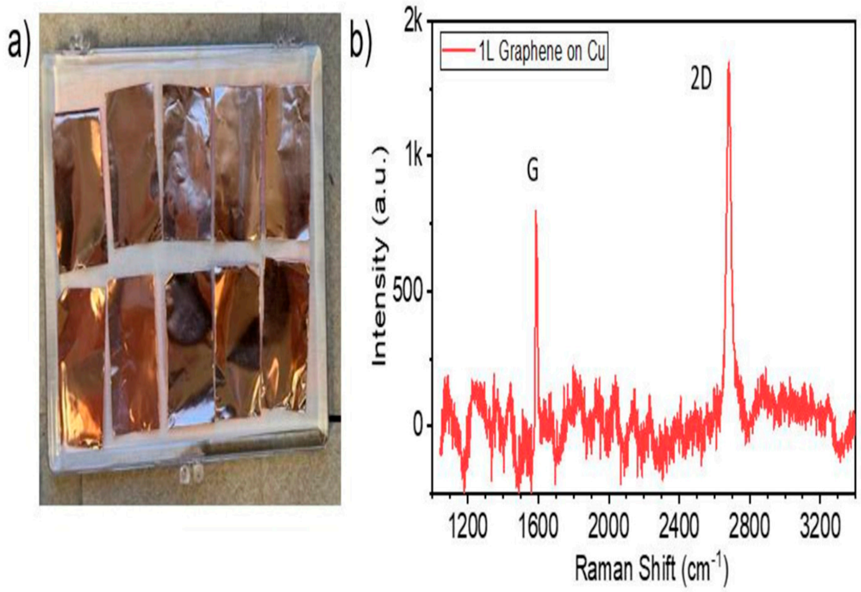

Figure S1: Raman spectra of monolayer graphene on a copper substrate

Table S1: LS4D media components

COMPONENTS	mM	Molar mass	g/L	Trace Mineral	per L	Thauers Vitamins	mg/L
Sodium Sulfate	50	142.04	7.102	Nitrilotriacetic acid	12.8g	Biotin	2
Sodium Lactate	60	112.06	6.7236	$FeCl_2.2H_2O$	1g	Folic acid	2
Magnesium Chloride	8	95.211	0.761688	$MnCl_2.4H_2O$	0.5g	Pyridoxine hydrochloride	10
Ammonium Chloride	20	53.491	1.06982	$CoCl_2.6H_2O$	0.3g	Thiamin hydrochloride	5
Dipotassium phosphate	2.2	174.2	0.38324	$ZnCl_2$	0.2g	Riboflavin	5
Calcium Chloride	0.6	110.98	0.066588	$Na_2MoO_4.2H_2O$	50mg	Nicotinic Acid	5
PIPES	30	302.4	9.072	H_3BO_3	20mg	DL-pantothenic acid	5

NaOH	10	39.997	0.39997	Vitamin B_{12}	0.1
	mL per Liter			p-aminobenzoic acid	5
Trace mineral solution	12.5	Autoclaved separately and added.		Lipoic acid	5
Thauers vitamin solution	1	Filter sterilized		Choline chloride	200
	µm		mg/L		
Resazurin	0.06	229.191	0.01375		

Table S2: Sequences of oligonucleotide primers used for qPCR

Gene		Forward Primer	Reverse Primer
HK	*Dde3717*	TTTAAAACAGCCTGGCCCGA	CAGCGCAAATTCCAGCACAT
PS	*Dde3253*	TCGCCGTGTTCGTGGTATTT	AAGCAGAAAGTCCATGCCGA
LuxP	*Dde3311*	CGGCTTTGCGTTATGCCTAC	CAAAACGCTTTGCTCCGTGA
RpoN	*Dde3097*	CACTGAAATCCGGCAAAGCC	GAATCTTCCGGCGCTTACCT
dsrB	*Dde0528*	TCGAGTCCAAATCCAAATCCA	GAATCTTCTTCACTTCGCGGG
Sat	*Dde2265*	CGCTGTTGAAGTGTGTGACG	CGTAGTTCTGGCGGAAGGTT
